# Humoral immune response and live-virus neutralization of the SARS-CoV-2 omicron (BA.1) variant after COVID-19 mRNA vaccination in children and young adults with chronic kidney disease

**DOI:** 10.1007/s00467-022-05806-9

**Published:** 2022-11-21

**Authors:** Maximilian Stich, Veronica Di Cristanziano, Burkhard Tönshoff, Lutz Thorsten Weber, Jörg Dötsch, Marian Theodor Rammer, Susanne Rieger, Eva Heger, Sven F. Garbade, Kathrin Burgmaier, Louise Benning, Claudius Speer, Sandra Habbig, Sophie Haumann

**Affiliations:** 1 Department of Pediatrics I, University Children’s Hospital Heidelberg, Heidelberg, Germany; 2 Institute of Virology, University Hospital Cologne and Faculty of Medicine, University of Cologne, Cologne, Germany; 3 Department of Pediatrics, University Hospital Cologne and Faculty of Medicine, University of Cologne, Cologne, Germany; 4 Faculty of Applied Healthcare Science, Deggendorf Institute of Technology, Deggendorf, Germany; 5 Department of Nephrology, University of Heidelberg, Heidelberg, Germany

**Keywords:** COVID-19, SARS-CoV-2, Transplantation, Pediatric nephrology, Coronavirus

## Abstract

**Background:**

Data on humoral immune response to standard COVID-19 vaccination are scarce in adolescent patients and lacking for children below 12 years of age with chronic kidney disease including kidney transplant recipients.

**Methods:**

We therefore investigated in this retrospective two-center study (DRKS00024668; registered 23.03.2021) the humoral immune response to a standard two-dose mRNA vaccine regimen in 123 CKD patients aged 5–30 years. A live-virus assay was used to assess the serum neutralizing activity against the SARS-CoV-2 omicron (BA.1) variant.

**Results:**

Children aged 5–11 years had a comparable rate and degree of immune response to adolescents despite lower vaccine doses (10 µg vs. 30 µg BNT162b2). Treatment with two (odds ratio 9.24) or three or more (odds ratio 17.07) immunosuppressants was an independent risk factor for nonresponse. The immune response differed significantly among three patient cohorts: 48 of 77 (62.3%) kidney transplant recipients, 21 of 26 (80.8%) patients on immunosuppressive therapy, and 19 of 20 (95.0%) patients with chronic kidney disease without immunosuppressive therapy responded. In the kidney transplant recipients, immunosuppressive regimens comprising mycophenolate mofetil, an eGFR of < 60 mL/min/1.73 m^2^, and female sex were independent risk factors for nonresponse. Two of 18 (11.1%) and 8 of 16 (50.0%) patients with an anti-S1-RBD IgG of 100–1411 and > 1411 BAU/mL, respectively, showed a neutralization activity against the omicron variant.

**Conclusion:**

A standard mRNA vaccine regimen in immunosuppressed children and adolescents with kidney disease elicits an attenuated humoral immune response with effective live virus neutralization against the omicron variant in approximately 10% of the patients, underlying the need for omicron-adapted vaccination.

**Graphical abstract:**

**Figure:**
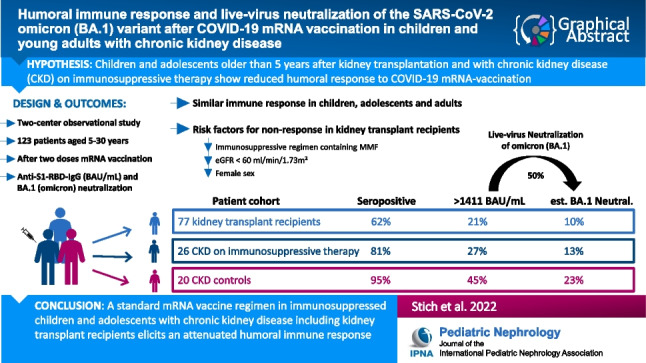
A higher resolution version of the Graphical abstract is available as Supplementary information

**Supplementary Information:**

The online version contains supplementary material available at 10.1007/s00467-022-05806-9.

## Introduction

Studies in adults have shown the attenuated immunogenicity of COVID-19 vaccines in patients on immunosuppressive medication including kidney transplant recipients (KTR) [1]. After a standard two-dose COVID-19 mRNA vaccination, both the rate of seroconversion and the magnitude of humoral immune response were lower than in healthy individuals [2]. A third and fourth dose of COVID-19 vaccine is currently recommended in these patients, because it substantially increases the rate of vaccine responders.

In the meantime, vaccination against COVID-19 was also approved for children and adolescents. However, data on vaccine response in adolescent patients with chronic kidney disease (CKD) on immunosuppressive therapy are scarce and mostly from single-center studies. To date, the available seven studies on adolescents comprise only 146 KTR with variable seropositivity rates between 44 and 86% [3–9]. None of them report data on neutralization activity against the BA.1 variant (omicron). Furthermore, data on vaccine response in immunosuppressed children aged 5–11 years only refer to four KTR [8] and four solid organ transplant recipients [9]. This age group is of particular interest as children younger than 12 receive 10 µg BNT162b2 vs. the 30 µg for older children and adolescents [10].

We therefore hypothesized that (i) the immune response after standard COVID-19 vaccination in children aged 5–11 years is inferior to that in adolescents and young adults, (ii) the immune response to standard COVID-19 vaccination in pediatric KTR and patients with CKD on immunosuppressive medication is attenuated compared to CKD patients without immunosuppressive medication, and (iii) the neutralization activity against the SARS-CoV-2 omicron variant using a live virus assay is only present in a minority of patients after standard COVID-19 vaccination.

## Methods

### Study design

This is a two-center retrospective observational cohort study to assess the humoral immune response including live virus neutralization against the SARS-CoV-2 omicron (BA.1) variant after standard COVID-19 mRNA vaccination in children, adolescents, and young adults with CKD at the University Children’s Hospitals in Heidelberg and Cologne, Germany. Inclusion criteria were the following: (i) CKD with or without immunosuppressive therapy, (ii) age ≥ 5 years, (iii) at least two vaccinations with an approved COVID-19 mRNA vaccine, and (iv) at least one analysis of SARS-CoV-2 antibodies reactive to the receptor-binding domain of the S1 glycoprotein (anti-S1-RBD) after the second vaccination. Vaccinations were performed according to the manufacturer’s recommendations regarding dose and time schedule (two vaccine doses within a 3-week interval). Study participants with a medical history of SARS-CoV-2 infection or antibodies against the nucleocapsid protein (indicative of previous SARS-CoV-2 infection) before or at the time of sample collection were excluded from the analysis.

Participants were recruited between April 2021 and April 2022. Blood was drawn at a median interval of 34 days (IQR 22.0–63.0) after administration of the second vaccine dose. As recommended, a third vaccine dose was proposed to all patients with failed seropositivity 4 weeks after two doses and in severely immunocompromised patients ≥ 12 years of age 6 months after the standard vaccination [11].

This study was approved by the respective ethics committees of the University of Heidelberg (S-201/2021) and Cologne (22–1242-retro) and conducted in accordance with the Declaration of Helsinki. The patients and/or their representative(s) gave informed written consent prior to participation, with consent or assent from patients as appropriate for their age. The study is registered at the German Registry for Clinical Studies (identifier: DRKS00024668). The study was designed, analyzed, and reported according to the STROBE guidelines (https://www.strobe-statement.org).

### Patients

Demographic and clinical data were extracted from electronical medical records. Kidney function was assessed by estimated glomerular filtration rate (eGFR) according to the modified Schwartz formula [12]. The intensity of immunosuppressive therapy was assessed by pediatric Vasudev score [13]. The following three patient cohorts were investigated: (i) KTR on immunosuppressive therapy, (ii) patients with CKD on immunosuppressive therapy, and (iii) patients with CKD without immunosuppressive medication. CKD is defined according to KDIGO as abnormalities of kidney structure or function present for > 3 months [14].

### Binding antibody assay

To analyze the serological response to COVID-19 vaccination, we measured the level of IgG binding the receptor-binding domain of the S1 glycoprotein (anti-S1-RBD). Blood samples were analyzed using the ADVIA Centaur sCOVG assay kit (11,207,377; Siemens) at the Siemens ADVIA Centaur in Heidelberg and the SARS-CoV-2 IgG II Quant assay (06S6132; Abbott) at Alinity I (Abbott, Abbott Park, IL, United States) in Cologne. The assays were performed, and cut-offs for seropositivity were defined according to the manufacturer’s instructions and as described previously [15, 16]. To convert measured indices to the WHO international standard binding antibody units per milliliter (BAU/mL), a factor of 21.8 (Heidelberg) and 0.142 (Cologne) was used. Thirty-six samples were analyzed with both assays, and the anti-S1-RBD-IgG level (BAU/mL) correlated between the assays (Spearman *r* = 0.98; 95% CI 0.96–0.99; *P* < 0.001).

### Live virus neutralization assay

In a sub-cohort of patients with an anti-S1-RBD IgG of ≥ 100 BAU/mL and sufficient serum available, the functional neutralizing activity against the omicron (BA.1) variant was tested using a live virus neutralization assay. For the neutralization assay, the virus was isolated from a nasal swab and expanded in a culture of VeroE6 cells by superinfection of VeroE6 from the initial outgrowth culture [17]. Whole genome sequencing of the isolated virus was performed through Illumina sequencing. The virus spike amino acid sequence is identical to the omicron variant BA.1.17.2 (EPI_ISL_13237863). As previously described [18], serum samples were serially diluted (1:10, 1:50, 1:250, 1:1250, 1:6250, and 1:31,250) and mixed with 100 TCID_50_ (50% tissue culture infectious dose) of live virus. The virus-serum mixture was incubated for 1 h at 37 °C. Thereafter, 50 μL of a Vero E6 cell suspension (250,000 cells/mL) was added to each sample dilution. The cells were incubated at 37 °C for 4 days before microscopically determining virus-related cytopathic effects (CPE) such as cell rounding, detachment, degeneration, and syncytium formation. Wells with a clear cytopathic effect of more than 10% of that of the virus control well (cells plus virus) were determined as positive (no detectable neutralizing activity). Wells with no CPE were classified as negative (detectable neutralizing activity).

### Statistical analysis

Statistical analyses were performed using SPSS 27 (IBM Corp., Armonk, NY, USA) and GraphPad Prism Version 9 (GraphPad Software, San Diego, CA, USA). Continuous variables are given as median and interquartile range (IQR). Binary or categorical variables are given as absolute (*n*) and relative frequencies (%). Differences in continuous variables between more than two groups were compared by Kruskal–Wallis test and between two groups including specific sample pairs for stochastic dominance by Mann–Whitney U test with Holm–Bonferroni correction. Differences in binary or categorical variables were assessed by chi-squared tests. To investigate the predictive value of risk factors for lacking seroconversion, a multiple binary logistic regression analysis, with the predictors age category (5.0–11.9, 12.0–17.9, and ≥ 18 years), eGFR (≤ or > 60 mL/min/1.73 m^2^), sex (male or female), and number of immunosuppressive agents (0, 1, 2, or ≥ 3) for the whole study cohort (model 1) and immunosuppressive regimen (calcineurin inhibitor [CNI] with [i] mycophenolate mofetil [MMF], [ii] azathioprine, [iii] everolimus, or [iv] CNI or everolimus with a steroid) for the subgroup analysis of KTR (model 2), was performed. No imputation was performed. The correlation of anti-S1-RBD IgG level to live virus neutralization titer was calculated with Spearman’s rho. A receiver operating characteristic (ROC) curve analysis was performed to define a cut-off anti-S1-RBD IgG level to predict any neutralization (ID_50_ ≥ 1:10) with at least 80% sensitivity and highest specificity possible. No a priori formulated hypotheses were tested; therefore, all *P* values and confidence intervals (CI) are reported as descriptive measures.

## Results

### Patient characteristics

**Fig. 1:**
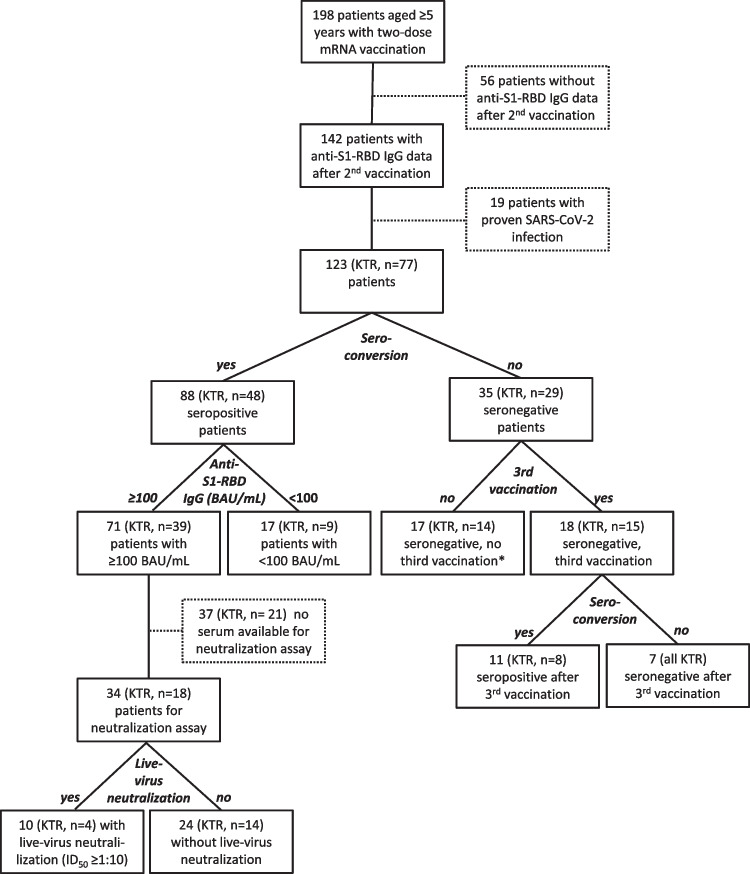
Disposition of study participants. *Five kidney transplant recipients experienced a RT-PCR-proven SARS-CoV-2 infection. BAU, binding antibody units; KTR, kidney transplant recipients; RBD, receptor binding domain

We included 123 patients (45 females, 36.6%) from two tertiary pediatric nephrology centers (Fig. 1). The median age was 14.1 years (range 5–30). Forty-three of the 123 patients (35.0%) were in the age category of 5–11 years with a median age of 8.5 years (IQR 7–10). All of them received the Biontech/Pfizer vaccine BNT162b2 (Comirnaty™) containing a dosage of 10 µg according to the manufacturer’s recommendations. The group of patients aged 12–17 years comprised 47 patients (38.2%) with a median age of 15.0 years (IQR 13–16); all received BNT162b2 (30 µg per injection). We included a group of 33 young adults with a median age of 20.4 years (IQR 19–23) treated in our pediatric centers; 30 received BNT162b2 (30 µg per injection), two patients received the Moderna vaccine elasomeran (Spikevax™, 100 µg per injection), and one patient received Biontech/Pfizer for their first dose and Moderna for the second dose. None of the patients had a serious adverse event (death, life-threatening hospitalization [initial or prolonged], disability or permanent damage, or required intervention to prevent permanent impairment or damage) as defined by the FDA [19].

Three patient cohorts were investigated: KTR (*n* = 77), patients with CKD on immunosuppressive medication due to other indications (*n* = 26), and, for comparison, patients with CKD without immunosuppressive therapy (*n* = 20). Details on patient characteristics including age, sex, eGFR, and immunosuppressive medication are given in Table 1.

**Table 1:** Patient characteristics

Characteristics	All patients *n* = 123	Kidney transplant recipients, *n* = 77	CKD on immunosuppressive therapy, *n* = 26	CKD controls *n* = 20
Sex, female, *n* (%)	45 (36.6)	28 (36.4)	10 (38.5)	7 (35.0)
Age, median (range)	14.1 (5–30)	15.1 (5–30)	13.0 (5–25)	12.5 (5–24)
Age group				
5.0–11.9 years, *n* (%)	43 (35.0)	23 (29.9)	11 (42.3)	9 (45.0)
12.0–17.9 years, *n* (%)	47 (38.2)	31 (40.3)	10 (38.5)	6 (30.0)
≥ 18 years, *n* (%)	33 (26.8)	23 (29.9)	5 (19.2)	5 (25.5)
eGFR (mL/min/1.73 m^2^), median (IQR)	63.4 (44.9–98.7)	56.0 (45.8–70.4)	109.5 (86.9–134.3)	48.8 (19.8–116.6)
eGFR				
> 60 mL/min/1.73 m^2^, *n* (%)	65 (52.8)	34 (44.2)	22 (84.6)	9 (45.0)
≤ 60 mL/min/1.73 m^2^, *n* (%)	58 (47.2)	43 (55.8)	4 (15.4)	11 (55.0)^a^
Primary kidney disease				
CAKUT, *n* (%)	39 (31.7)	34 (44.2)	0	5 (25.0)
Cystic kidney disease, *n* (%)	21 (17.1)	19 (24.7)	0	2 (10.0)
Glomerular disease, *n* (%)	45 (36.6)	15 (19.5)	20 (76.9)	10 (50.0)
Vasculitis	3 (2.4)	0	3 (11.5)	0
Others, *n* (%)	15 (12.2)	9 (11.7)	3 (11.5)	3 (15.0)
Immunosuppressive therapy, number of agents				
0, *n* (%)	20 (16.3)	0	0	20 (100)
1, *n* (%)	21 (17.1)	0	21 (80.8)^c^	0
2, *n* (%)	47 (38.2)	43 (55.8)^b^	4 (15.4)^d^	0
≥ 3, *n* (%)	35 (28.5)	34 (44.2)^b^	1 (3.8)^e^	0
SARS-CoV-2 seropositive				
Yes No	88 (71.5) 35 (28.5)	48 (62.3) 29 (37.7)	21 (80.8) 5 (19.2)	19 (95.0) 1 (5.0)

^a^CKD stage 5 on chronic dialysis therapy (hemodialysis, *n* = 2; peritoneal dialysis, *n* = 1); CKD stage 4, *n* = 4; CKD stage 3, *n* = 4

^b^Details on immunosuppressive medication in kidney transplant recipients are given in Table 3

^c^Mycophenolate mofetil (MMF), *n* = 11; steroids, *n* = 3; rituximab during 6 months prior to vaccination, *n* = 3; tacrolimus (Tac), *n* = 2; ciclosporin, *n* = 1; eculizumab, *n* = 1

^d^Steroids in conjunction with Tac, *n* = 2, or azathioprine, *n* = 1, or MMF, *n* = 1

^e^Steroids in conjunction with Tac and MMF, *n* = 1

*CAKUT*, congenital anomalies of the kidney and urinary tract; *CKD*, chronic kidney disease; *eGFR*, estimated glomerular filtration rate. No missing data for presented variables

### Humoral immune response against standard COVID-19 mRNA vaccination and risk factors for non-response

**Fig. 2:**
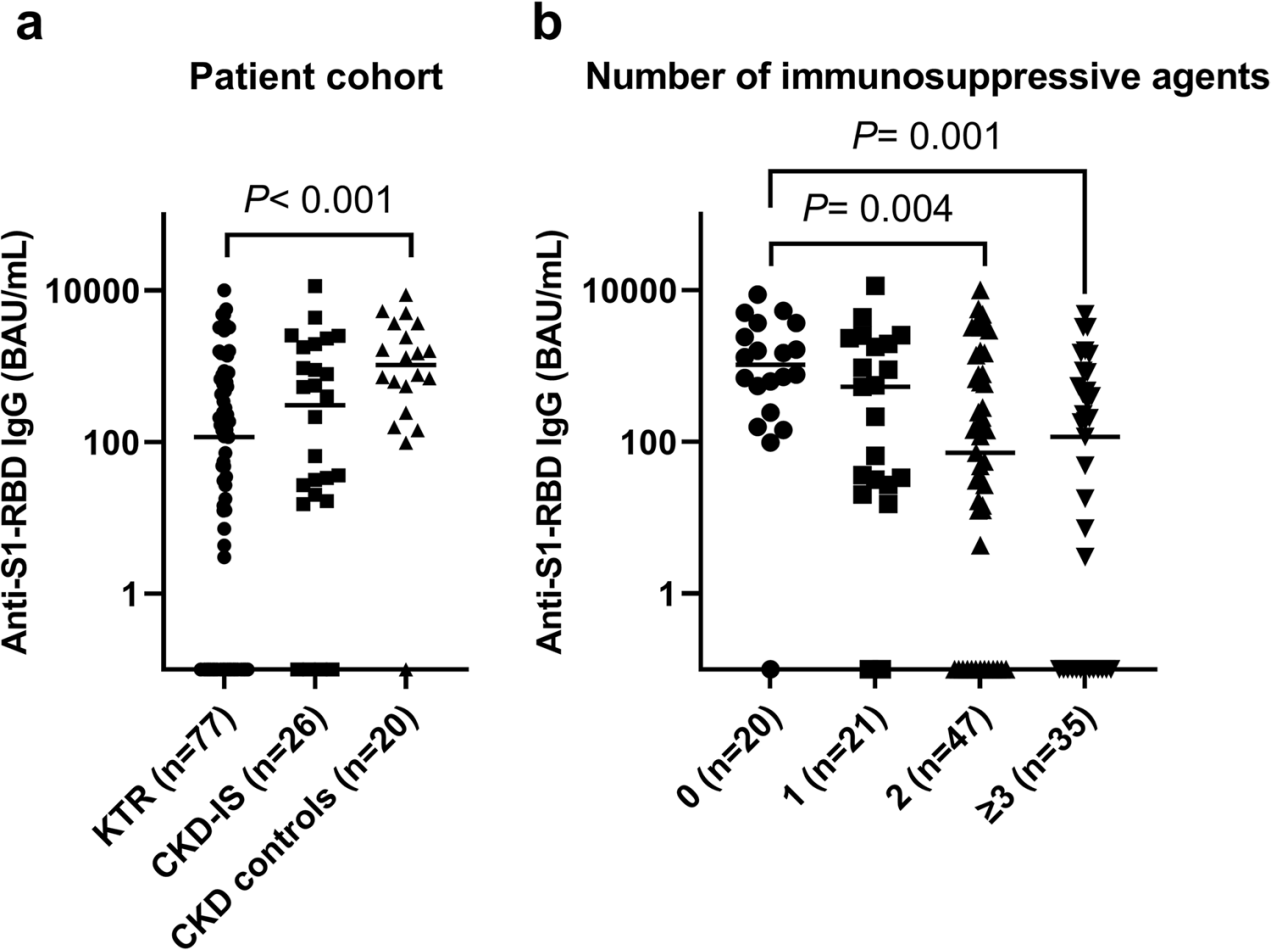
Anti-SARS-CoV-2 S1-RBD-IgG (BAU/mL) on a log_10_-scale after a standard two-dose COVID-19 mRNA vaccine regimen. Panel **a** data stratified according to the three patient cohorts and panel **b** data stratified according to the number of immunosuppressive agents. The respective median is indicated by a straight line. *P*-values are calculated using a Mann–Whitney U-test with Holm–Bonferroni correction and are only displayed when *P* < 0.1. KTR, kidney transplant recipients; CKD-IS, patients with chronic kidney disease on immunosuppressive therapy; CKD, patients with chronic kidney disease without immunosuppressive therapy

A positive SARS-CoV-2 anti-S1-RBD antibody response after a standard two-dose mRNA vaccine regimen was detected in 88 patients (71.5%) (Fig. 1). The time interval between the second vaccination and blood sampling was not different between responders and nonresponders (34.5 [IQR 22.0–65.5] days vs*.* 31.0 [IQR 20.5–42.5] days, *P* = 0.473). The humoral immune response differed significantly (*P* = 0.008) among the three patient cohorts: 48 of 77 (62.3%) KTR, 21 of 26 (80.8%) patients with CKD on immunosuppressive medication, and 19 of 20 (95%) patients with CKD without immunosuppressive medication responded (Table 1). The only nonresponder in the CKD cohort was a patient on hemodialysis with multiple co-morbidities (microcephalic osteodysplastic primordial dwarfism type 1). The anti-S1-RBD IgG level was ninefold lower (*P* < 0.001) in KTR (117 [IQR 0–769] BAU/mL) as compared to the patients with CKD without immunosuppressive medication (1046 [IQR 470–2735] BAU/mL) (Fig. 2a).

We included the following four factors with a potential impact on anti-SARS-CoV-2 seropositivity in the multiple binary logistic regression analysis: age category, sex, eGFR, and number of immunosuppressive agents (Table 2). Children aged 5–11 years receiving a vaccine dose of 10 µg BNT162b2 had a comparable rate and degree of immune response as adolescents and young adults receiving a vaccine dose of 30 µg BNT162b2. Compared to the patients with CKD without immunosuppressive medication (no immunosuppressive therapy), treatment with two (OR 9.24; 95% CI 1.61–175.7) or three or more immunosuppressive agents (OR 17.07; 95% CI 2.91–328.0) was an independent risk factor for nonresponse, and the magnitude of humoral immune response was impaired in these patients (Fig. 2b).

**Table 2:** Multiple binary logistic regression analysis on the entire patient cohort to identify independent risk factors for humoral nonresponse to standard COVID-19 mRNA vaccination

Patient characteristics	SARS-CoV-2 antibody response		Odds ratio (95% confidence interval)	*P*-value
	Positive *n* = 88	Negative *n* = 35		
Age group				0.301
5.0–11.9 years, *n* (%)	32 (36.4)	11 (31.4)		
12.0–17.9 years, *n* (%)	36 (40.9)	11 (31.4)	0.65 (0.22 – 1.87)	0.427
≥ 18 years, *n* (%)	20 (22.7)	13 (37.1)	1.52 (0.52–4.48)	0.438
Sex				0.089
Male, *n* (%)	59 (67.0)	19 (54.3)		
Female, *n* (%)	29 (33.0)	16 (45.7)	2.17 (0.89–5.42)	
eGFR				0.169
> 60 mL/min/1.73 m^2^, *n* (%)	51 (58.0)	14 (40.0)		
≤ 60 mL/min/1.73 m^2^, *n* (%)	37 (42.0)	21 (60.0)	1.89 (0.77–4.78)	
Immunosuppressive therapy				0.007
0 agents, *n* (%)	19 (21.6)	1 (2.9)		
1 agent, *n* (%)	18 (20.5)	3 (8.6)	3.76 (0.41 – 82.8)	0.282
2 agents, *n* (%)	32 (36.4)	15 (42.9)	9.24 (1.61–175.7)	0.040
≥ 3 agents, *n* (%)	19 (21.6)	16 (45.7)	17.07 (2.91–328.0)	0.009

*eGFR*, estimated glomerular filtration rate. No missing data for presented variables

### Subgroup analysis on kidney transplant recipients

**Fig. 3:**
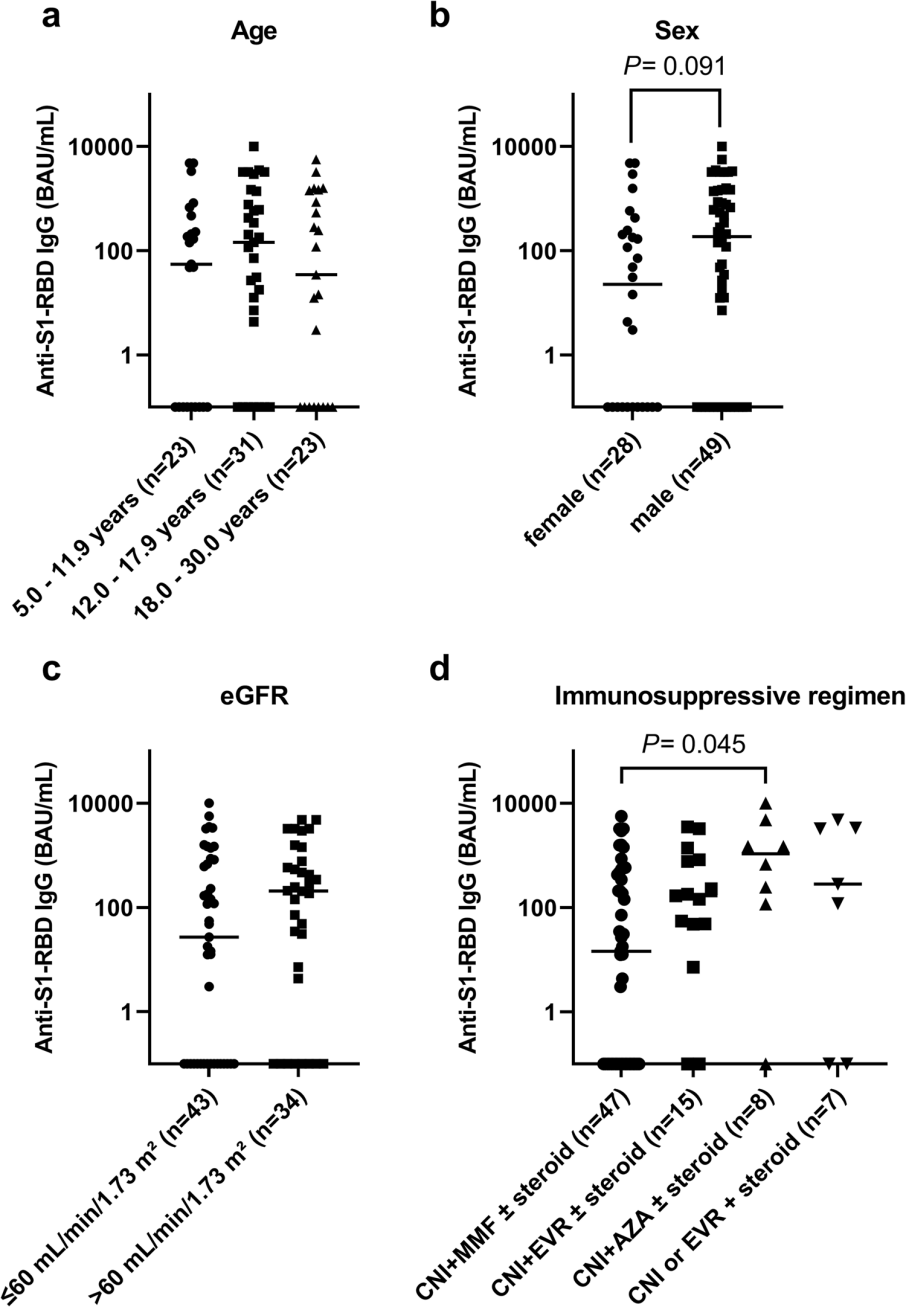
Subgroup analysis of anti-SARS-CoV-2 S1-RBD IgG (BAU/ml) in kidney transplant recipients (KTR) after standard SARS-CoV-2 vaccination according to age category (**a**), sex (**b**), eGFR (**c**), and immunosuppressive regimen (**d**) on a log_10_-scale. The median is indicated by a straight line. *P*-values are calculated using a Mann–Whitney U-test with Holm–Bonferroni correction and are only displayed when *P* < 0.1

An analysis of the subgroup of 77 KTR confirmed that seroconversion (Table 3) and relative anti-S1-RBD IgG levels (Fig. 3a) were not significantly different among age categories. Transplantation-related factors (donor source and time since transplantation) or primary kidney diseases were not associated with significantly different seroconversion rates (Table 3).

**Table 3:** Patient characteristics of kidney transplant recipients and humoral immune response to a standard two-dose COVID-19 mRNA vaccine regimen

Patient characteristics	All patients *n* = 77	SARS-CoV-2 antibody response		*P*-value
		Positive *n* = 48 (62.3%)	Negative *n* = 29 (37.7%)	
Sex, female, *n* (%)	28 (36.4)	14 (29.2)	14 (48.3)	0.091
Age group				0.691
5.0–11.9 years, *n* (%)	23 (29.9)	14 (29.2)	9 (31.0)	
12.0–17.9 years, *n* (%)	31 (40.3)	21 (43.8)	10 (34.5)	
≥ 18 years, *n* (%)	23 (29.9)	13 (27.1)	10 (34.5)	
Primary kidney disease				0.122
CAKUT, *n* (%)	34 (44.2)	20 (41.7)	14 (48.3)	
Cystic kidney disease, *n* (%)	19 (24.7)	16 (33.3)	3 (10.3)	
Glomerular disease, *n* (%)	15 (19.5)	8 (16.7)	7 (24.1)	
Others, *n* (%)	9 (11.7)	4 (8.3)	5 (17.2)	
Donor source				0.682
Living donor, *n* (%)	27 (35.1)	16 (33.3)	11 (37.9)	
Deceased donor, *n* (%)	50 (64.9)	32 (66.7)	18 (62.1)	
Time since transplant, years				0.566
< 3, *n* (%)	20 (26.0)	12 (25.0)	8 (27.6)	
3–10, *n* (%)	40 (51.9)	27 (56.3)	13 (44.8)	
> 10, *n* (%)	17 (22.1)	9 (18.8)	8 (27.6)	
eGFR (mL/min/1.73 m^2^) median (IQR)	55.8 (44.9–70.4)	60.1 (46.3–79.9)	51.6 (41.0–63.0)	0.145
eGFR				0.184
> 60 mL/min/1.73 m^2^, *n* (%)	34 (44.2)	24 (50.0)	10 (34.5)	
≤ 60 mL/min/1.73 m^2^, *n* (%)	43 (55.8)	24 (50.0)	19 (65.5)	
Intensity of immunosuppression				
Pediatric VASUDEV score, median (IQR)	4.3 (3.3–5.7)	4.2 (3.2–5.6)	4.6 (3.5–7.0)	0.219
Immunosuppressants				
Tacrolimus, *n* (%)	65 (84.4)	40 (83.3)	25 (86.2)	0.736
No tacrolimus, *n* (%)	12 (15.6)	8 (16.7)	5 (13.8)	
Ciclosporin, *n* (%)	8 (10.4)	5 (10.4)	3 (10.3)	0.992
No ciclosporin, *n* (%)	69 (89.6)	43 (89.6)	26 (89.7)	
MMF, *n* (%)	47 (61.0))	24 (50.0))	23 (79.3)	0.011
No MMF, *n* (%)	30 (39.0))	24 (50.0)	6 (20.7)	
Everolimus, *n* (%)	19 (24.7)	15 (31.3)	4 (13.8)	0.085
No everolimus, *n* (%)	58 (75.3)	33 (68.8)	25 (86.2)	
Azathioprine, *n* (%)	8 (10.4)	7 (14.6)	1 (3.4)	0.121
No azathioprine, *n* (%)	69 (89.6)	41 (85.4)	28 (96.6)	
Glucocorticoids, *n* (%)	41 (53.2)	23 (47.9)	18 (62.1)	0.228
No glucocorticoids, *n* (%)	36 (46.8)	25 (52.1)	11 (37.9)	
Immunosuppressive regimens				0.074
CNI + MMF ± steroid, *n* (%)	47 (61.0)	24 (50)	23 (79.3)	
CNI + EVR ± steroid, *n* (%)	15 (19.5)	12 (25.0)	3 (10.3)	
CNI + AZA ± steroid, *n* (%)	8 (10.4)	7 (14.6)	1 (3.4)	
CNI or EVR + steroid, *n* (%)	7 (9.1)	5 (10.4)	2 (6.8)	

The *P* value refers to a comparison between seropositive and seronegative patients

*AZA*, azathioprine; *CAKUT*, congenital anomalies of the kidney and urinary tract; *CNI*, calcineurin inhibitor; *eGFR*, estimated glomerular filtration rate; *EVR*, everolimus; *MMF*, mycophenolate mofetil. No missing data for presented variables

In the multiple binary logistic regression analysis (Table 4), which included the potential risk factors age category, sex, eGFR, and immunosuppressive regimen, female sex was associated with a 3.11-fold (95% CI 1.05–10.0, *P* = 0.046) higher risk for lacking humoral immune response. An eGFR ≤ 60 mL/min/1.73m^2^ increased the risk of humoral nonresponse by a factor of 3.44 (95% CI 1.16–11.4, *P* = 0.032). The different immunosuppressive regimens were also identified as independent risk factors (*P* = 0.041) for lacking a vaccine response: patients on a CNI-based immunosuppressive regimen in conjunction with MMF (with or without steroids) had a higher risk than patients on a CNI-regimen in conjunction with everolimus (with or without steroids) (OR 0.15; 95% CI 0.02–0.72, *P* = 0.029) or azathioprine (with or without steroids) (OR 0.09; 95% CI 0.01–0.62, *P* = 0.037). The magnitude of humoral immune response was numerically lower in females compared to males (22.8 [IQR 0–236.9] BAU/mL vs. 186.4 [IQR 0–1411] BAU/mL, *P* = 0.091) (Fig. 3b) and significantly lower in patients on a CNI-based immunosuppressive regimen in conjunction with MMF compared to patients on a CNI-based immunosuppressive regimen in conjunction with azathioprine (14.6 [IQR 0–470.6] BAU/mL vs*.* 1081.4 [IQR 149.7–3990] BAU/mL, *P* = 0.045) (Fig. 3d).

**Table 4:** Multiple binary logistic regression analysis on kidney transplant recipients to identify independent risk factors for humoral nonresponse to standard COVID-19 mRNA vaccination

Patient characteristics	SARS-CoV-2 antibody response		Odds ratio (95% confidence interval)	*P*-value
	Positive *n* = 48	Negative *n* = 29		
Age group				0.452
5.0–11.9 years, *n* (%)	14 (29.2)	9 (31.0)		
12.0–17.9 years, *n* (%)	21 (43.8)	10 (34.5)	0.42 (0.10–1.58)	0.207
≥ 18 years, *n* (%)	13 (27.1)	10 (34.5)	0.58 (0.13–2.42)	0.458
Sex				0.046
Male, *n* (%)	34 (70.8)	15 (51.7)		
Female, *n* (%)	14 (29.2)	14 (48.3)	3.11 (1.05–10.0)	
eGFR				0.032
> 60 mL/min/1.73 m^2^, *n* (%)	24 (50.0)	10 (34.5)		
≤ 60 mL/min/1.73 m^2^, *n* (%)	24 (50.0)	19 (65.5)	3.44 (1.16–11.4)	
Immunosuppressive regimen				0.041
CNI + MMF ± steroid, *n* (%)	24 (50)	23 (79.3)		
CNI + EVR ± steroid, *n* (%)	12 (25.0)	3 (10.3)	0.15 (0.02–0.72)	0.029
CNI + AZA ± steroid, *n* (%)	7 (14.6)	1 (3.4)	0.09 (0.01–0.62)	0.037
CNI or EVR + steroid, *n* (%)	5 (10.4)	2 (6.8)	0.22 (0.02–1.46)	0.144

*AZA*, azathioprine; *CNI*, calcineurin inhibitor; *eGFR*, estimated glomerular filtration rate; *EVR*, everolimus; *MMF*, mycophenolate mofetil. No missing data for presented variables

### Impact of a third vaccination on humoral immune response in kidney transplant recipients

Of the 35 humoral nonresponders after the second vaccination, 29 were KTR, of whom 15 received a third vaccination (Fig. 1). After the third vaccination, eight of 15 (53.3%) previous nonresponders (seronegative KTR) mounted antibodies against SARS-CoV-2, but the magnitude of humoral immune response was lower than in those with a response after two vaccinations (anti-S1-RBD IgG 120.1 [IQR, 50.8–340.6] BAU/mL vs*.* 506.2 [IQR 144.7–1583] BAU/mL, *P* = 0.036) (Supplementary Fig. 1). The seven patients without a humoral immune response after three vaccinations either received an immunosuppressive regimen containing MMF or had an inherited T-cell deficiency (Supplementary Table 1).

We also evaluated the impact of a third vaccine dose on participants with a positive humoral response after the second vaccine dose. Of 56 humoral responders ≥ 12 years of age, 30 (53.6%) received a third vaccination, of whom 25 (83.3%) were KTR (median age 16.8 [IQR 15–18] years). Twenty-three of 25 (92.0%) KTR mounted a higher immune response after the third compared to the second vaccine dose with a median 3.4-fold (IQR 1.7–6.1) increase (anti-S1-RBD IgG 2617 [IQR 621.2–4629] BAU/mL vs. 586.6 [IQR 180.9–1573] BAU/mL, *P* < 0.001) (Supplementary Fig. 2).

### Neutralization against SARS-CoV-2 omicron (BA.1) variant

**Fig. 4:**
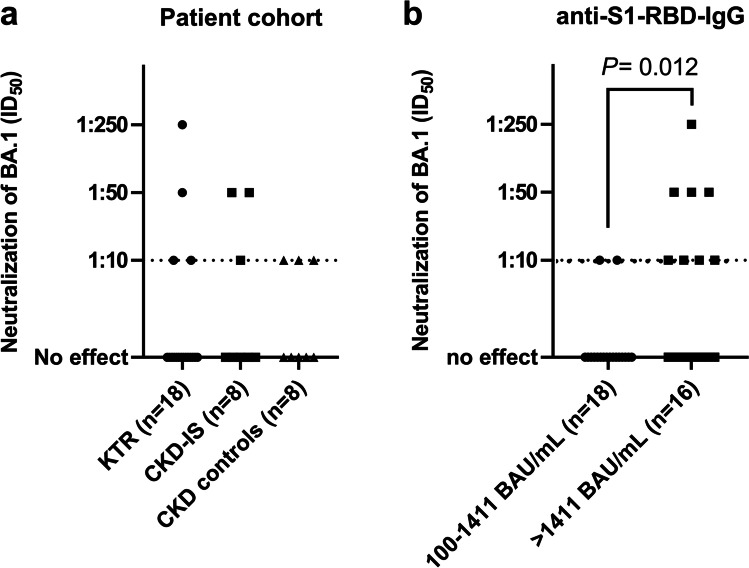
Live-virus neutralization of BA.1 (omicron) stratified by patient cohort (**a**) and anti-S1-RBD IgG level of 100–1411 BAU/mL and > 1411 BAU/mL (**b**) on a log_10_-scale with a cut-off for this assay of 1:10. *P*-values are calculated using a Mann–Whitney U-test with Holm–Bonferroni correction and are only displayed when *P* < 0.1. KTR, kidney transplant recipients; CKD-IS, patients with chronic kidney disease on immunosuppressive therapy; CKD, patients with chronic kidney disease without immunosuppressive therapy

In 34 patients with a BAU of ≥ 100/mL and available serum samples, we determined the serum neutralizing activity against the SARS-COV-2 omicron (BA.1) variant using a live virus neutralization assay (Fig. 1). Ten of the 34 patients (29.4%) showed a neutralizing activity against the omicron variant. Any live virus neutralization was detected in 4 of 18 (22.2%) KTR, 3 of 8 (37.5%) patients with CKD on immunosuppressive therapy, and 3 of 8 (37.5%) patients with CKD without immunosuppressive medication (Fig. 4a). The majority (60.0%) of patients with neutralizing activity showed neutralization at the lowest dilution of 1:10. Neutralizing activity at a higher dilution (1:250) was identified in only one patient who also had the highest anti-S1-RBD IgG of 10,077 BAU/mL. There was a significant positive correlation between the anti-SARS-CoV-2-IgG titer and the corresponding neutralizing activity (*R* = 0.714, *P* < 0.001).

A ROC curve analysis of relative anti-SARS-CoV-2 S1-RBD IgG levels for discrimination of neutralizing serum samples (ID_50_ ≥ 1:10) was performed to define a relative anti-S1-RBD IgG level that has at least 80% sensitivity and highest possible specificity to predict the neutralization of the omicron variant (ROC-AUC 0.74; 95% CI 0.54–0.95, *P* = 0.028) (Supplementary Fig. 3). The optimal cut-off was 1411 BAU/mL (sensitivity 80%, specificity 66.7%). Fifty percent of the patients above and only 11% of the patients below this cut-off had any neutralization activity; relative neutralization titers differed significantly (*P* = 0.012) between the two groups (Fig. 4b).

As live virus neutralization was only determined in patients with a BAU of ≥ 100/mL, we extrapolated the finding from the ROC curve analysis to the entire patient cohort (*n* = 123) (Table 1). We assumed that an anti-S1-RBD-IgG level > 1411/mL (entire cohort [*n* = 32], KTR [*n* = 16], CKD with [*n* = 7] and without [*n* = 9] immunosuppressive medication) is associated with neutralization activity in 50% of the patients. Hence, approximately 13.0% of the entire patient cohort and 10.4% of KTR, 13.5% of the patients with CKD on immunosuppressive medication, and 22.5% of the patients with CKD without immunosuppressive medication can be estimated to have a serum neutralizing activity against the omicron variant after a standard two-dose mRNA vaccine regimen.

## Discussion

The main result of this study is that a standard COVID-19 mRNA vaccine regimen in immunosuppressed pediatric KTR and CKD patients elicits an attenuated humoral immune response, which confirms our hypothesis: only 62.3% of KTR and 80.8% of the CKD patients on immunosuppressive therapy did respond compared to 95% of the patients with CKD without immunosuppressive medication. Also, the magnitude of humoral immune response in KTR was ninefold lower than in the patients with CKD without immunosuppressive medication; treatment with two or three or more immunosuppressants was an independent risk factor for nonresponse. The seropositivity rate of 62.3% in KTR is comparable to the pooled data on seropositivity rate of 57.5% from seven published studies (*n* = 146 KTR) on adolescents and young adults [3–9]. It is higher than the pooled seropositivity rate of 45% in older adult solid organ transplant recipients (mean age > 50 years) [1]. Hence, young age appears to be associated with better humoral immune response to COVID-19 mRNA vaccination.

So far, there is only one study that published data on 4 KTR [8] and one study on 4 solid organ transplant recipients under the age of 12 years [9]. The current study is the first study that describes the humoral immune response to a standard two-dose COVID-19 mRNA vaccine regimen in children with a functioning kidney transplant in comparison with CKD with and without immunosuppressive therapy below the age of 12 years. In contrast to our hypothesis, we observed that these patients have a comparable rate and degree of immune response as the adolescents and young adults. The younger children received a vaccine dose (10 µg BNT162b2) threefold lower than that administered to the adolescents and adults (30 µg BNT162b2). However, whether this also applies for omicron-adapted booster vaccinations cannot be easily extrapolated and should be evaluated.

Risk factors for nonresponse to a standard two-dose COVID-19 mRNA vaccine regimen were the number of immunosuppressive agents and, in KTR, an immunosuppressive regimen containing MMF. The latter observation is consistent with the data obtained in adolescent [4, 7] and adult [20–27] KTR. It is currently being investigated in adults whether the transient halt of MMF therapy increases the rate of vaccine responders without an undue risk of kidney allograft rejection [28, 29]. However, severe COVID-19 in pediatric patients on immunosuppressive therapy is rare [30–32]. This is especially true for the currently predominant omicron variants that are associated with less severe disease than the previous variants [33]. Whether MMF withdrawal might also play a role in pediatric populations depends on the further evolution of the pandemic.

Interestingly, in our study, female sex was associated with increased risk of humoral nonresponse (OR 3.11; 95% CI 1.02–9.48). This observation is consistent with the finding of a recent meta-analysis [1] on adults that females have a slightly increased risk of nonresponse to a standard two-dose COVID-19 mRNA vaccine regimen. The precise biological mechanism of this sex difference remains to be elucidated.

The correlate of protection after COVID-19 vaccination is currently being debated [34, 35]. Commercially available serological assays to determine seropositivity and relative anti-SARS-CoV-2 IgG levels are used in clinical practice to determine seroconversion and humoral immune response to guide the decision on which patients at risk should receive additional vaccinations beyond the standard vaccine regimen. These assays were designed to detect antibodies directed against the SARS-CoV-2 wild-type strain; the currently used cut-off values for seropositivity do not reflect the actual protection against variants of concern. We therefore analyzed serum live virus neutralization against the SARS-CoV-2 omicron (BA.1) variant in a subset of patients. We observed that only a small subset of children and adolescents with CKD showed any live virus neutralization against omicron. An anti-S1-RBD-IgG of > 1411/mL was associated with at least 50% probability of any neutralization activity. When extrapolating these data to the entire patient cohort, approximately 10% of KTR, 13% of the patients with CKD on immunosuppressive medication, and 23% of the patients with CKD without immunosuppressive medication can be estimated to have a serum neutralization activity against the omicron variant after a standard two-dose mRNA vaccine regimen. This is the first study that reports data on live virus neutralization in children after kidney transplantation or on immunosuppressive therapy due to other chronic kidney diseases. The data are consistent with observations on adult KTR that the majority of humoral immune responders insufficiently neutralize variants of concern and especially omicron even after a third COVID-19 vaccination [36, 37]. Therefore, an omicron-adapted vaccination regimen is urgently needed. An adapted BA.4/5 spike protein bivalent booster vaccine was authorized by the FDA [38] and EMA [39] in August and September 2022 for adults and children older than 12 years, and children aged 5–11 years were included by the FDA in October [40].

In our study, approximately 50% of previous nonresponders showed a humoral immune response to a third vaccination. The magnitude of humoral immune response was lower than in those with a response after two vaccinations. A recent meta-analysis on adult solid organ transplant recipients reported a pooled seroconversion rate of 63% after the third vaccine dose compared to the 45% after the second dose [1]. Strategies to optimize vaccine response in nonresponders are currently being evaluated on adults. They may comprise the combination of different vaccine types to a heterologous vaccination regimen, for example, vector vaccine priming followed by mRNA vaccine boosting [41–43]. However, non-mRNA vaccines are also based on SARS-CoV-2 wild-type and not recommended and approved for children.

Our study has limitations. Although live virus neutralization assays are considered the current gold standard to determine actual neutralization titers and test results correlate well with protection from SARS-CoV-2 infections [44], absolute cut-off values for protection from symptomatic infection or severe disease especially for new emerging variants have not been established for most assays including ours. We cannot exclude the possibility of asymptomatic infections for those infected during the vaccination schedule and especially for those with failed seroconversion or seroreversion of anti-nucleocapsid antibodies. Given such high rates of prior SARS-CoV-2 infection we are now seeing, it will be interesting to study in the future the vaccine response in those with prior infection. Another limitation is missing data on cellular immunity. Some studies on KTR observed T-cell responses to be developing in greater frequency than humoral responses [41, 45, 46]. However, T-cell assays are less standardized among different laboratories and more resource-intensive, and the clinical interpretation is difficult. Recent studies on healthy subjects [47] and patients on immunosuppressive medication [48, 49] including kidney [23, 50] and liver [51] transplant recipients have shown a strong correlation between anti-S1-RBD antibodies and SARS-CoV-2-specific IFN-γ and functional T-cell responses. Thus, it appears feasible to use anti-S1-RBD IgG data as a surrogate parameter for assessment of immunity after COVID-19 vaccination.

In conclusion, a standard mRNA vaccine regimen in pediatric KTR and children and adolescents with CKD on immunosuppressive therapy elicits an attenuated humoral immune response. Children aged 5–11 years receiving a lower vaccine dose have a comparable rate and degree of immune response as adolescents. Risk factors for nonresponse comprise the number of immunosuppressive agents and, in KTR, a regimen containing MMF, an eGFR < 60 mL/min/1.73 m^2^, and female sex. Live virus neutralization against the omicron variant is achieved in approximately 10% of pediatric CKD patients with immunosuppression. This underlines the need for omicron-adapted bivalent booster vaccination for adults and children ≥ 5 years of age, as currently recommended by the CDC [52].

## Supplementary Information

Below is the link to the electronic supplementary material.

Graphical Abstract (PPTX 59.5 KB)

Supplementary file2 (DOCX 261 KB)

## Data Availability

De-identified data will be made available upon publication to researchers who provide a methodologically sound proposal for use in achieving the goals of the approved proposal. Proposals should be submitted to sandra.habbig@uk-koeln.de.
