# Supplementary material for: Humoral immune response and live-virus neutralization of the SARS-CoV-2 omicron (BA.1) variant after COVID-19 mRNA vaccination in children and young adults with chronic kidney disease
Source: Pediatr Nephrol. 2022 Nov 21;38(6):1935–48. doi: 10.1007/s00467-022-05806-9 (PMC9684918; doi:10.1007/s00467-022-05806-9)
Supplement: Supplementary file 1 — Graphical Abstract (PPTX 59.5 KB) [file 467_2022_5806_MOESM1_ESM.pptx]

## Slide 1
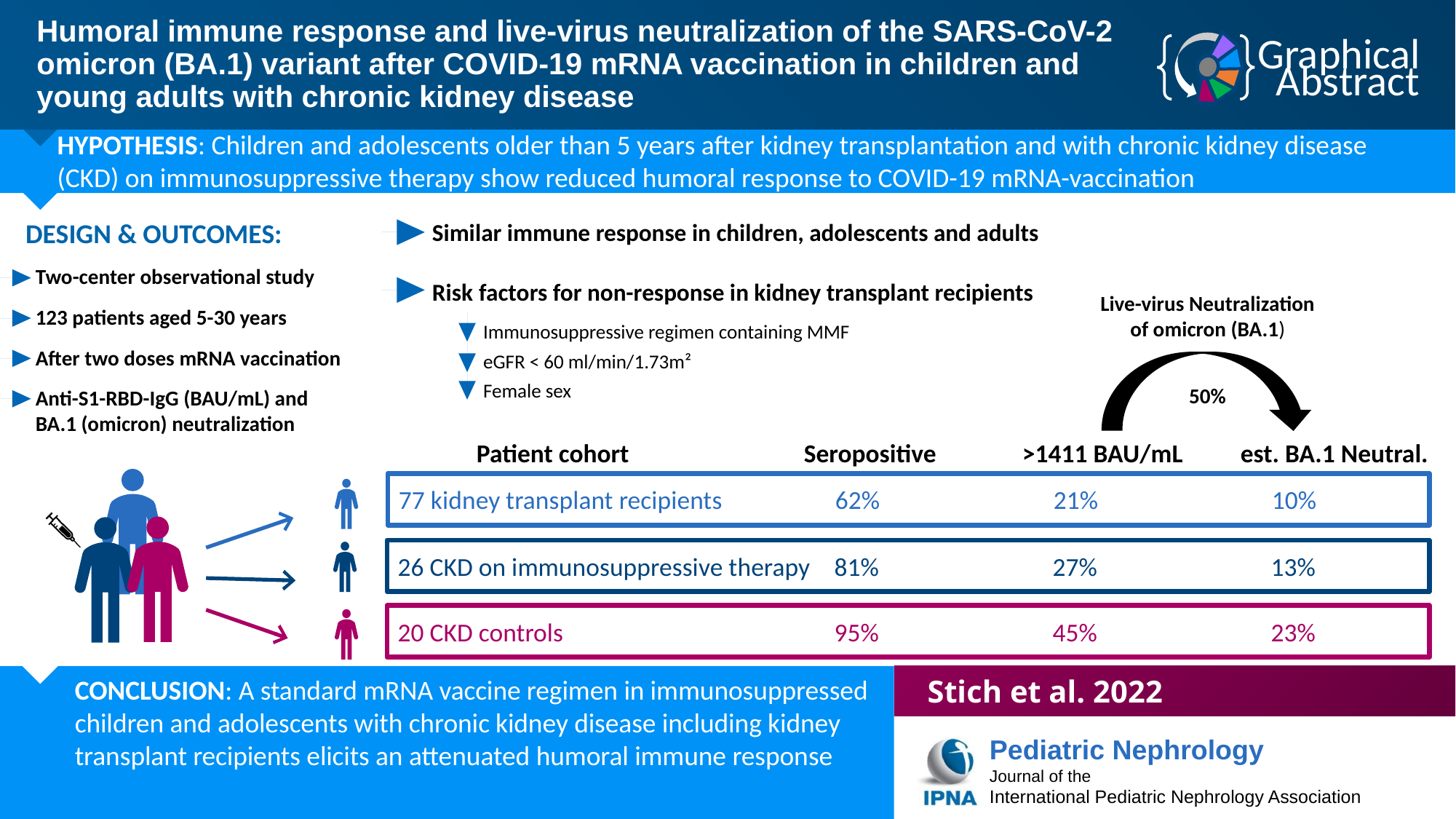

Humoral immune response and live-virus neutralization of the SARS-CoV-2 omicron (BA.1) variant after COVID-19 mRNA vaccination in children and
young adults with chronic kidney disease
HYPOTHESIS: Children and adolescents older than 5 years after kidney transplantation and with chronic kidney disease (CKD) on immunosuppressive therapy show reduced humoral response to COVID-19 mRNA-vaccination
DESIGN & OUTCOMES:
Similar immune response in children, adolescents and adults
Risk factors for non-response in kidney transplant recipients
Two-center observational study
123 patients aged 5-30 years
After two doses mRNA vaccination
Anti-S1-RBD-IgG (BAU/mL) and
BA.1 (omicron) neutralization
Live-virus Neutralization of omicron (BA.1)
Immunosuppressive regimen containing MMF
eGFR < 60 ml/min/1.73m²
Female sex
50%
	Patient cohort		Seropositive	>1411 BAU/mL	est. BA.1 Neutral.
77 kidney transplant recipients 	62%		21%		10%
26 CKD on immunosuppressive therapy	81%		27%		13%
20 CKD controls	 		95%		45%		23%
CONCLUSION: A standard mRNA vaccine regimen in immunosuppressed children and adolescents with chronic kidney disease including kidney transplant recipients elicits an attenuated humoral immune response
Stich et al. 2022
