# Supplementary material for: Humoral immune response and live-virus neutralization of the SARS-CoV-2 omicron (BA.1) variant after COVID-19 mRNA vaccination in children and young adults with chronic kidney disease
Source: Pediatr Nephrol. 2022 Nov 21;38(6):1935–48. doi: 10.1007/s00467-022-05806-9 (PMC9684918; doi:10.1007/s00467-022-05806-9)
Supplement: Supplementary file 2 — Supplementary file2 (DOCX 261 KB) [file 467_2022_5806_MOESM2_ESM.docx]

**Electronic supplementary material**

**Supplementary Table 1** Clinical characteristics of kidney transplant recipients with lacking humoral immune response after three vaccinations with mRNA vaccine

| **Patient** | **Sex** | **Primary kidney disease** | **Time period between transplantation and vaccination (years)** | **Age at vaccination (years)** | **Vaccine** | **Immunosuppressive medication** |
| --- | --- | --- | --- | --- | --- | --- |
| 1 | f | Schimke immuno-osseous dysplasia | 9 | 22 | BNT162b2  (30 µg) | Tacrolimus  Prednisone |
| 2 | m | ALAD porphyria | 11 | 29 | BNT162b2  (30 µg) | Tacrolimus  MMF  Prednisone |
| 3 | m | CAKUT | 3 | 7 | BNT162b2 (10 µg) | Tacrolimus  MMF  Prednisone |
| 4 | m | CAKUT | 15 | 19 | BNT162b2 (30 µg) | Tacrolimus  MMF  Prednisone  Rituximab |
| 5 | f | aHUS | 13 | 16 | BNT162b2 (30 µg) | Tacrolimus  MMF  Prednisone |
| 6 | m | CAKUT | 8 | 16 | BNT162b2 (30 µg) | Tacrolimus  MMF |
| 7 | f | SRNS and FSGS | 12 | 24 | BNT162b2 (30 µg) | Tacrolimus  MMF |

aHUS, atypical hemolytic uremic syndrome; ALAD, delta-aminolevulinic acid dehydratase; CAKUT, congenital anomalies of the kidney and urinary tract; FSGS, focal-segmental glomerulosclerosis, MMF, mycophenolate mofetil; SRNS, steroid-resistant nephrotic syndrome

**
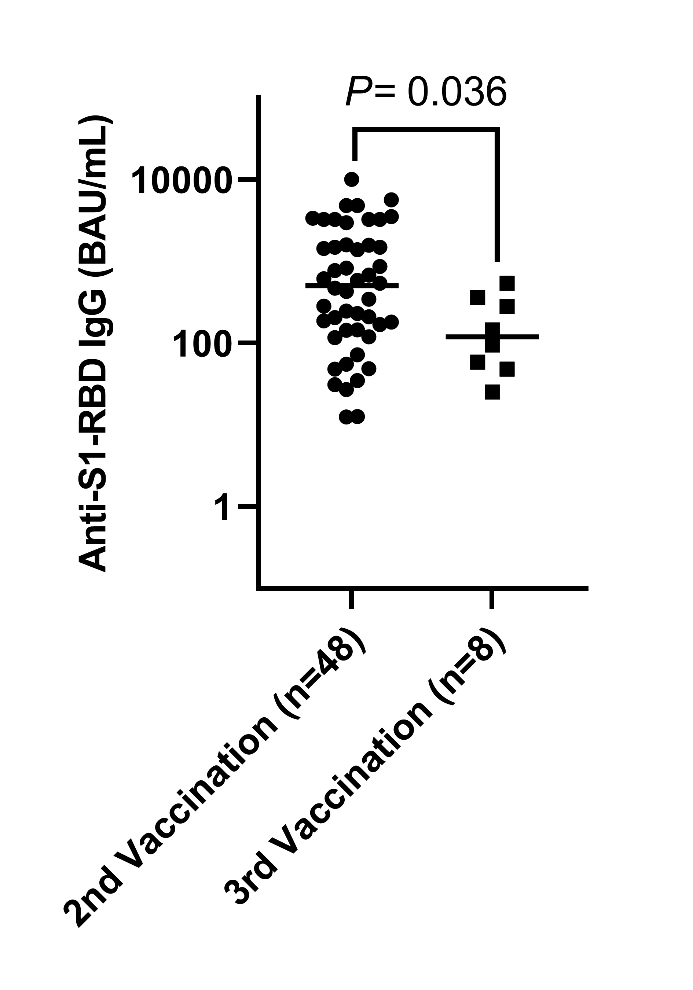
**

**Supplementary Figure 1** Comparison of anti-SARS-CoV-2-S1-RBD IgG level (BAU/mL, log_10_-scale) in kidney transplant recipients (KTR) who seroresponded after two vs. three vaccine doses. Median is given as a straight line. *P*-value was calculated using a Mann-Whitney-U-test with Holm-Bonferroni correction.

**
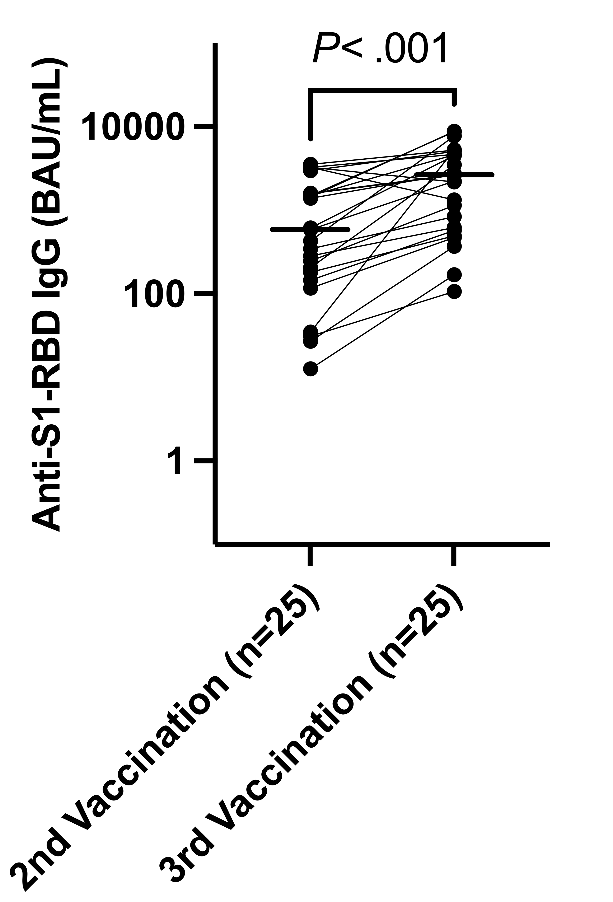
**

**Supplementary Figure 2** Longitudinal anti-SARS-CoV-2-S1-RBD IgG level (BAU/mL, log_10_-scale) after the second and third vaccine dose in 25 kidney transplant recipients (KTR) with a positive seroresponse after the second vaccine dose. Median is given as a straight line. *P-*value was calculated using a Wilcoxon matched-pairs signed rank test.

**
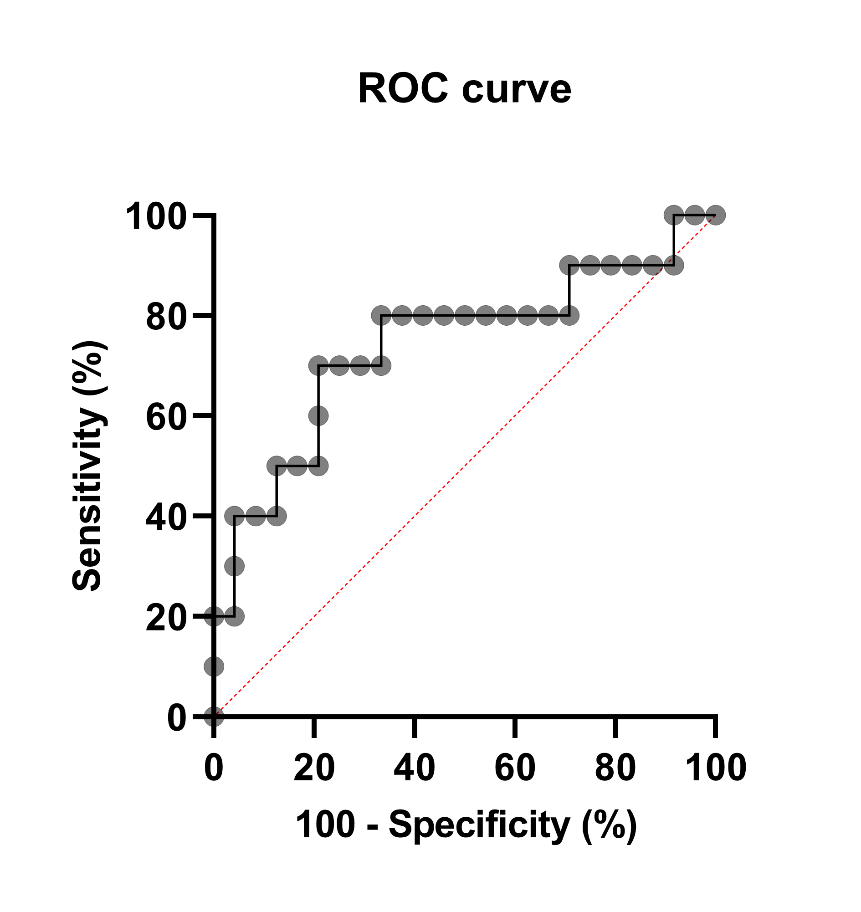
**

**Supplementary Figure 3** Receiver operating characteristic (ROC) curve analysis of relative anti-SARS-CoV-2 S1-RBD IgG levels for discrimination of neutralizing serum samples (ID_50_ ≥1:10) [ROC-AUC, 0.74; 95% CI, 0.54 – 0.95, *P* = 0.028].
